# The establishment and application of a dual Nano-PCR detection method for feline calicivirus and feline herpesvirus type I

**DOI:** 10.3389/fmicb.2023.1285268

**Published:** 2023-11-16

**Authors:** Manping Yan, Jinyuan Shang, Xiaohao Zhang, Shun Wu, Chunxia Wang, Zhenjun Wang, Guoliang Luo, Li Yi, Xiaofeng Shan, Yuening Cheng, Erkai Feng

**Affiliations:** ^1^Key Laboratory of Economic Animal Diseases, Ministry of Agriculture, Institute of Special Animal and Plant Sciences, Chinese Academy of Agricultural Sciences, Changchun, China; ^2^College of Animal Science and Technology, Jilin Agricultural University, Changchun, China; ^3^Department of Cardiology, The Second Hospital of Jilin University, Changchun, China

**Keywords:** feline calicivirus, feline herpesvirus type I, Nano-PCR, detection diagnostic technology, infectious disease

## Abstract

Feline calicivirus (FCV) and Feline herpesvirus type I (FHV-I) are the main pathogens causing upper respiratory tract infections in cats, and some wild animals. These two viruses always coinfection and cause serious harm to pet industry and wild animals protection. Established a rapid and accurate differential diagnosis method is crucial for prevention and control of disease, however, the current main detection method for these two viruses, either is low sensitivity (immunochromatographic strip), or is time-consuming and cannot differential diagnosis (conventional single PCR). Nanoparticle-assisted polymerase chain reaction (Nano-PCR) is a recently developed technique for rapid detection method of virus and bacteria. In this study, we described a dual Nano-PCR assay through combining the nanotechnology and PCR technology, which for the clinical simultaneous detection of FCV and FHV-I and differential diagnosis of upper respiratory tract infections in cats or other animals. Under optimized conditions, the optimal annealing temperature for dual Nano-PCR was 51.5°C, and specificity test results showed it had no cross reactivity to related virus, such as feline panleukopenia virus (FPV), feline Infectious peritonitis virus (FIPV) and rabies virus (RABV). Furthermore, the detection limit of dual Nano-PCR for FCV and FHV-I both were 1 × 10^−8^ ng/μL, convert to number of copies of virus DNA was 6.22 × 10^3^copies/μL (FCV) and 2.81 × 10^3^copies/μL (FHV-I), respectively. The dual Nano-PCR detected result of 52 cat clinical samples, including ocular, nasal and faecal swabs, and (3 FCV-positive samples), was consistent with ordinary PCR and the clinical detection results. The dual Nano-PCR method established in this study with strong specificity and high sensitivity can be used for virus nucleic acid (FCV and FHV-I) detection of clinical samples of feline upper respiratory tract infections feline calicivirus and feline herpesvirus while providing support for the early diagnosis of cats that infected by FCV and FHV-I.

## 1. Introduction

As the main pathogen causing upper respiratory tract infections in cats and some wild animals such as lions, tigers, and leopards, the clinical characterization of FHV-I and FCV is almost identical, include respiratory inflammation, ocular inflammation, hair loss around the eyes, and thick secretions from the eyes and nose. Therefore, it is nearly impossible to distinguish the viruses based on clinical symptoms. Most of the time, laboratory nucleic acid detection methods are needed. In these methods, specific PCR amplification technology plays an important role in differential diagnosis (Bo et al., 2023; Jindong et al., 2023; Ying et al., 2023).

FCV, belongs to the order of *Picornavirales* and the family of *Caliciviridae*, is a nonenveloped single-stranded positive-sense RNA virus with a genome length of approximately 7,700 bp and three open reading frames (ORFs) (Kai et al., 2017). The ORF1 encodes 7 non-structural proteins, and ORF2 and ORF3 encode the major structural protein VP1 and the minor structural protein VP2, respectively. VP1 is encoded by the conserved region of the gene sequence, and the presence of the virus is often determined by identifying the VP1 fragment. FCV is highly contagious and can cause mild to severe respiratory and oral disease in cats. Most strains are nonlethal, but a few are lethal. FCV is easily mutated, so FCV vaccines do not completely protect against the mutated strains (Zhanding et al., 2019; Chengyun et al., 2023; Weijie et al., 2023).

FHV-I, also known as viral rhinobronchitis, is a member of the Var*icellovirus* genus of the herpesvirus subfamily *Alphaherpesvirina*. It is an enveloped, double-stranded DNA virus with a genome length of approximately 126–135 kb with 78 open reading frames that can encode 74 kinds of proteins. Currently, only one serotype of the virus has been identified. FHV-I replicates and proliferates on the conjunctiva, respiratory epithelial cells, and neuronal cells. The virus mainly infects domestic cats and cats such as lions, tigers and leopards and has a mortality rate of up to 50%. FHV-I is different from FCV in that its survivability is relatively low, and it is sensitive to organic solvents such as acid, ether, and chloroform. It can be killed by common disinfectants, and it does not survive at high temperatures (Meihui et al., 2023; Weijie et al., 2023; Xinyan et al., 2023).

Currently, the detection method of FCV and FHV-I is based on the initial judgment of clinical symptoms, or using test strips but the sensitivity is very low, and the early stage of the disease cannot be detected. There is also a method of virus isolation, but the efficiency is low and time-consuming.

Nanoparticle-assisted polymerase chain reaction (Nano-PCR) is a recently developed technique for rapid detection method of virus and bacteria (Jianke et al., 2015). Gold nanoparticles can promote the combination of primers and templates by increasing the thermal conductivity of the PCR, which can improve the specificity and sensitivity of the detection method (Lin et al., 2017). In 2019, Qin T, et al. established nanoparticle-assisted PCR (Nano-PCR) assay for the detection of canine parvovirus (CPV), and in 2022, Jingfei et al. published the Nano-PCR detection method of feline panleukopenia virus (FPV) (Tong et al., 2019; Jingfei et al., 2022). All of these just detect one pathogen, and not-simultaneous detection of FCV and FHV-I and differential diagnosis of upper respiratory tract infections in cats or other animals.

Therefore, in this study, we described a dual Nano-PCR method, which can specifically detect FHV-I and FCV at the same time and it will provide important technical support for the prevention of animal diseases in the pet industry. Meanwhile, this study also provides more sensitive and efficient detection methods for the timely diagnosis and prevention of other wild protected animal diseases susceptible to FCV and FHV-I.

## 2. Materials and methods

### 2.1. Sources of experimental materials

FCV, FHV-I, FPV, Rabies virus (RABV) cDNA and FIPV were isolated and preserved by the Institution of Special Animals and Plant Sciences, Chinese Academy of Agricultural Sciences (CAAS). Gold nanoparticles were purchased from Shiao Biotechnology Co., Ltd. (Changchun, Jilin). EasyPure® Viral DNA/RNA Kit was purchased from TransGen Biotech (Beijing, China). RevertAid Master Mix was purchased from ThermoFisher Scientific. Plasmid Mini Kit I and Gel Extraction Kit were purchased from Omega. pMD™ 19-T Vector Cloning Kit was purchased from Takara (Beijing). 2 × M5 HiPer plus Taq HiFi PCR mix (with blue dye) was purchased from Beijing Jumei Biotechnology Co., Ltd.

### 2.2. Experimental instruments

A high-speed centrifuge was purchased from Thermo Fisher Scientific (China) Co., Ltd.; a nanophotometer was purchased from Implen GmbH; a Multi-Temp Platform was purchased from Monad Biotech Co. Ltd.; a gene amplification instrument was purchased from Beijing Dinghaoyuan Technology Co., Ltd.

### 2.3. Design primers

According to the gene sequences of FCV strains and FHV-I strains in GenBank, primers were designed using Oligo7.0 and Primer5.0, and the primers were synthesized by Jilin Province Kumei Biotechnology Co., Ltd. Refering to Table 1 for details.

**Table 1:** Primers for the dual Nano-PCR method.

Primer	Primer sequences (5′-3′)	Segment length (bp)	Target gene
FCV-F	TAGATATGGGTTTAGAAGGG	242	VP1
FCV-R	TTAGATTGGAAGCGGATG		
FHV-I-F	AGATTTGCCGCACCATACCTTC	518	TK
FHV-I-R	CCGGGCTTTGAAAACACTGAAT		

### 2.4. Viral nucleic acid extraction

The FCV and FHV-I viruses frozen at −80°C were thawed, and the RNA of FCV and the DNA of FHV-I were extracted using the EasyPure ® Viral DNA/RNA Kit purchased from TransGen Biotech. The DNA was stored at −20°C, and the RNA was obtained from Thermo Scientific RevertAid Master Mix and reverse transcribed into cDNA. The remaining RNA was stored at −80°C, and the obtained cDNA was stored at −20°C.

### 2.5. Construction of FCV- and FHV-I-positive plasmids

Using the extracted FCV cDNA and FHV-I DNA as templates, the primer sequences designed in 2.3 were used to amplify the target fragments, gel recovery and purification of the target fragments were then performed, and they were connected to the pMD™ 19-T vector. Technology Co., Ltd. provided sequencing services and finally FCV-positive plasmids and FHV-I-positive plasmids were obtained.

### 2.6. Establishment of a common dual PCR method for FCV and FHV-I

The FCV- and FHV-I-positive plasmids obtained in 2.5 were used as templates for PCR amplification. Reaction system (20 μL): 10 μL 2 × M5 HiPer plus Taq HiFi PCR mix (with blue dye); 0.5 μL FCV-F, 0.5 μL FCV-R, 0.5 μL FHV-I-F, 0.5 μL FHV-I-R; 0.25 μL template; volume to 20 μL. The reaction conditions were as follows: predenaturation at 95°C for 3 min; denaturation at 94°C for 25 s, annealing at 53°C for 25 s, and extension at 72°C for 10s for a total of 35 cycles; and extension at 72°C for 5 min. PCR amplification products were detected by agarose gel electrophoresis.

### 2.7. Establishment of a dual Nano-PCR detection method for FCV and FHV-I

#### 2.7.1. Optimization of nanoparticle size and dosage

Gold Nanoparticles of different sizes (30, 50, 70, 100 nm) were used sequentially. There was a total of four groups; each group used different amounts of Nanoparticles (0.5, 1.0, 1.5, 2.0 μL). PCR system (20 μL): 10 μL 2 × M5 HiPer plus Taq HiFi PCR mix (with blue dye); 0.5 μL FCV-F, 0.5 μL FCV-R, 0.5 μL FHV-I-F, 0.5 μL FHV-I-R, respectively; different amounts (0.5, 1.0, 1.5, 2.0 μL) of Nano gold particles (30, 50, 70, 100 nm) were added, and 0.25 μL of the template was added, respectively. The mix was volumed up to 20 μL with water. The reaction conditions were predenaturation at 95°C for 3 min; denaturation at 94°C for 25 s, annealing at 53°C for 25 s, 72°C for 10s, for a total of 35 cycles; and 72°C for 5 min. PCR amplification products were detected by agarose gel electrophoresis.

#### 2.7.2. Optimization of annealing temperature

The FCV- and FHV-I-positive plasmids obtained in 2.5 were used as templates, the annealing temperature gradient was set at 50.0–60.0°C, and the other reaction conditions were the same. Nano-PCR system (20 μL): 10 μL 2 × M5 HiPer plus Taq HiFi PCR mix (with blue dye); 0.5 μL FCV-F, 0.5 μL FCV-R, 0.5 μL FHV-I-F, 0.5 μL FHV-I-R; 0.25 μL template; volume up to 20 μL with water. The reaction conditions were as follows: predenaturation at 95°C for 3 min; denaturation at 94°C for 25 s, annealing at 50.0–60.0°C (12 annealing temperatures in total) for 25 s, and extension at 72°C for 10s for a total of 35 cycles; and extension at 72°C for 5 min. PCR amplification products were detected by agarose gel electrophoresis.

#### 2.7.3. Specificity test

The optimized dual Nano-PCR detection method for FCV and FHV-I was used to detect the DNA or cDNA of FCV and FHV-I, FCV, FHV-I, FPV, FIP, and RABV to verify the specificity of the method.

#### 2.7.4. Sensitivity test

The concentrations of FCV- and FHV-I-positive plasmids determined by the Nanophotometer were 165.20 ng/μL and 159.45 ng/μL, respectively, and the converted copy numbers were 6.22 × 10^11^copies/μL and 2.81 × 10^11^copies/μL, respectively. ddH_2_O was used to dilute the two groups of positive plasmids according to a 10-fold ratio, and the plasmids of each dilution were used as templates to perform dual Nano-PCRs and common dual-PCRs. The products were subjected to agarose gel electrophoresis, and the electrophoresis bands were compared.

### 2.8. Clinical sample testing

Fifty-two cat eye, nose and faecal swabs were collected from different regions in Changchun City virual DNA and RNA were extracted by using Takara Mini BEST Viral RNA/DNA Extraction Kit Ver.5.0 (Takara, Beijing China) extracting. The RNA was reverse transcribed into cDNA. The DNA and cDNA of samples were detected by the established common Nano-PCR and dual Nano-PCR.

## 3. Results

### 3.1. Establishment of a common dual PCR method for FCV and FHV-I

**Figure 1:**
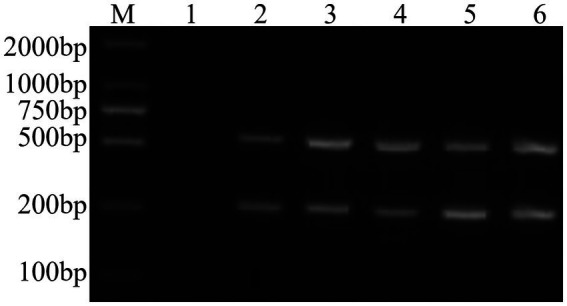
Establishment of a common dual PCR method. M: DNA molecular quality standard; 1: negative control; 2: positive control; 3–6: FCV- and FHV-I common dual PCR.

Through PCR amplification of the target fragments of FCV- and FHV-I-positive plasmids, after detection by agarose gel electrophoresis, the expected target fragments of 242 bp and 518 bp were obtained, and the ordinary dual PCR detection experiment was successfully established, as shown in Figure 1.

### 3.2. Establishment of the FCV and FHV-I dual Nano-PCR method

**Figure 2:**
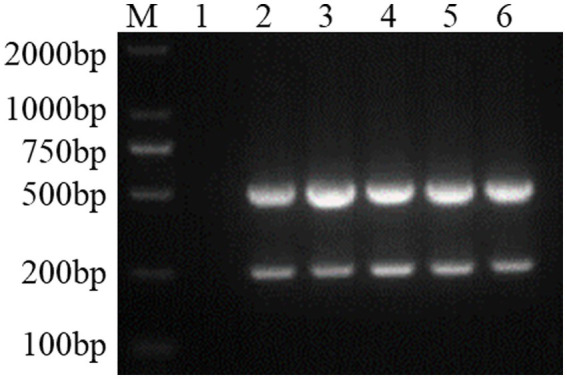
Establishment of a dual Nano-PCR method. M: DNA molecular quality standard; 1: negative control; 2: positive control; 3–6: FCV- and FHV-I dual Nano-PCR.

Using the dual Nano-PCR system, the target fragments of FCV- and FHV-I-positive plasmids were amplified, and after agarose gel electrophoresis, target fragments with expected sizes of 242 bp and 518 bp were obtained, as shown in Figure 2.

#### 3.2.1. Optimization of nanoparticle size and dosage

**Figure 3:**
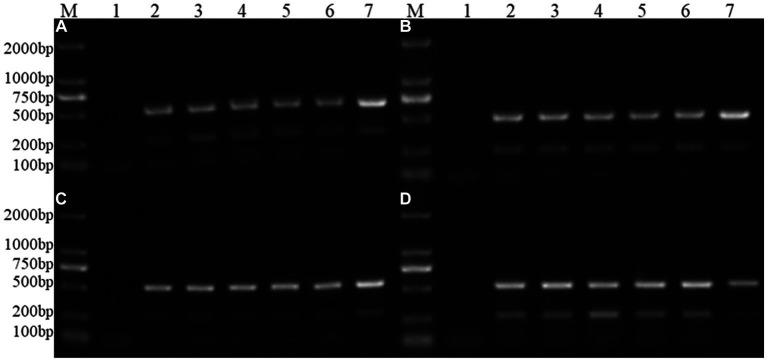
Optimization of nanoparticle size and dosage. M: DNA molecular quality standard; 1: Negative control; 2; 25 nm; 3: 30 nm; 4: 50 nm; 5: 70 nm; 6: 100 nm; 7: Positive control; 1 μL; **(A)**: 0.5 μL of nanoparticle; **(B)**: 1.0 μL of nanoparticle; **(C)**: 1.5 μL of nanoparticle; **(D)**: 2.0 μL of nanoparticle.

By simultaneously controlling the nanometre size as an independent variable and the amounts of nanoparticles as an independent variable, and keeping other conditions unchanged, the results of PCR products detected by agarose gel electrophoresis are shown in Figure 3. It can be seen from the figure that the target fragment is the brightest when the nanoparticle size is 50 nm and the amount added is 2 μL, so this condition is the final result of optimizing the nanoparticle size and dosage.

#### 3.2.2. Optimization of annealing temperature

**Figure 4:**
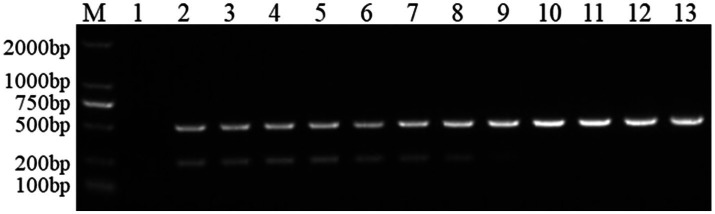
Optimization of annealing temperature. M: DNA molecular quality standard; 1: negative control; 2: 50.0°C; 3: 50.4°C; 4: 51.0°C; 5: 52.0°C; 6: 53.2°C; 7: 54.0°C; 10: 57.0°C; 11: 58.0°C; 12: 59.5°C; 13: 60.0°C.

Figure 4 shows the results of agarose gel electrophoresis after the FCV- and FHV-I-positive plasmids underwent an annealing temperature gradient (50.0–60.0°C) PCR. It can be seen from the figure that the target band is the clearest when the annealing temperature is 51.0°C and 52.0°C, so the final optimal annealing temperature was 51.5°C.

#### 3.2.3. Specificity test

**Figure 5:**
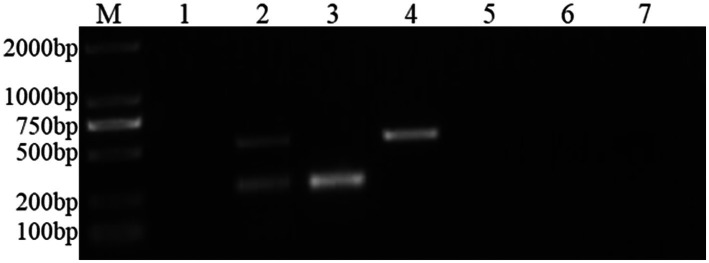
Specificity experiment of the dual Nano-PCR method. M: DNA molecular quality standard; 1: negative control; 2: FCV and FHV-I; 3: FCV; 4: FHV-I; 5: FPV; 6: FIPV; 7: RABV.

Bands of 242 bp and 518 bp appeared in the amplification results of FCV and FHV-I viruses using the established dual Nano-PCR method; only 242 bp bands appeared in the detection results of FCV virus; and only 518 bp bands appeared in the detection results of FHV-I virus. There were no bands in the detection of other pathogens, and the results are shown in Figure 5. It can be seen that the established dual Nano-PCR method has good specificity.

#### 3.2.4. Sensitivity experiment

**Figure 6:**
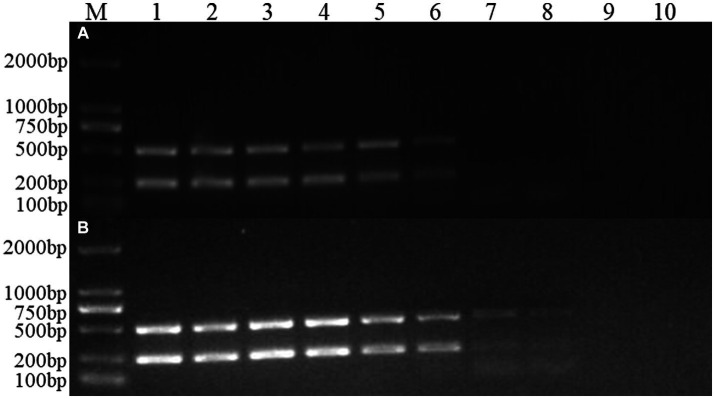
Sensitivity experiment of the dual Nano-PCR method. M: DNA molecular quality standard; 1–9: Plasmid dilutions are 10^−1^, 10^−2^, 10^−3^, 10^−4^, 10^−5^, 10^−6^, 10^−7^, 10^−8^, 10^−9^; 10: negative control; **(A)**: double ordinary PCR; **(B)**: dual Nano-PCR.

FCV- and FHV-I-positive plasmids were diluted 10 times to 10^−1^, 10^−2^, 10^−3^, 10^−4^, 10^−5^, 10^−6^, 10^−7^, 10^−8^, and 10^−9^. Each dilution was used as a template, and dual Nano-PCR and ordinary PCR experiments were carried out at the same time. It can be seen from Figure 6 that the dual Nano-PCR still had bands at 1 × 10^−8^ ng/μL, which was two orders of magnitude higher than the sensitivity of ordinary PCR.

### 3.3. Clinical sample testing

Using the dual Nano-PCR method established in this study to detect viruses in a total of 52 cat eye, nose and faecal swabs obtained from different regions in Changchun City, three bands were detected at 242 bp, the positive rate of FCV was 5.8%, and there were no cases of FHV-I. This result was consistent with the ordinary PCR detection established in 2.6.

## 4. Discussion

FCV was first isolated from the gastrointestinal tract of cats in New Zealand in 1957 and FCV vaccines have been used for more than 40 years, but outbreaks still occur, which due to the strong mutation ability of FCV. Sometimes, feline calicivirus virulent systemic disease (FCV-VSD), with higher mobility was always record in many contries (Caringella et al., 2019), FHV-I is a double-stranded DNA virus with stable structure. FHV-I rarely mutates, and it can detoxify intermittently and easy to lose activity. Therefore, it is not easy to cause large-scale outbreaks and the detection rate is low, but 80% of cats will carry it for life after infection (Yuzhen et al., 2023). Therefore, in this study, only 3 FCV strains were detected in 52 clinical samples, and no FHV-I strains were detected.

Among various detection technologies for viral pathogens such as FCV and FHV-I, quantitative real-time PCR (qPCR) can achieve relative quantification, with high sensitivity. However, it takes longer, costs more, and experimental operation is strict. It can only detect one pathogen at a time. In recent years, FCV and FHV-I have shown a trend of clustering in domestic cats or Feline animals, which has seriously threatened to the health of felines and the prevalence of families with pets (Huahua, 2021; Kingyan et al., 2022). The dual Nano-PCR detection method of FCV and FHV-I established in this study is convenient and fast, and the optimized annealing temperature is approximately 4°C lower than that of common Nano-PCR (Jun and Chunhai, 2007). This method has the advantages of thermostabilising the surface of DNA polymerase, adjusting the stability of DNA polymerization and reaction, reducing the Tm value, improving the amplification efficiency and having higher sensitivity and specificity than traditional PCR methods. According to the brightness of the strips under different nanometre diameters, it can be inferred that the brightness and diameter have a normal distribution trend, the optimum size is reached at approximately 50 nm, and the amplification efficiency is the highest at this time. Nanocarriers have advantages for basic research in the field of traumatic brain injury. This is worthy of indepth research (Jain et al., 2021; Sajinia and Roger, 2021; Xingshuang et al., 2023).

In this study, the dual Nano-PCR established also has some limitations. Although this method can simultaneously detect two pathogens causing upper respiratory tract infections, its sensitivity may be lower than that of a single detection (Sailike et al., 2021). Although we have bound nanoparticles, there may still be efficiency issues in primer substrate binding under both single and simultaneous reaction conditions, making it difficult to achieve a lower detection limit. We hope to further enhance reaction sensitivity by combining other chemical or biological materials that can promote the reaction in the future.

In conclusion, the dual Nano-PCR method of FCV and FHV-I established in this study has good specificity, and high sensitivity, and the minimum detection amount can reach 10^−8^ ng/μL. Early diagnosis of FCV and FHV-I, which present similar clinical characteristics, high infection rates, and easy spread provides important diagnostic support. This method provides important testing methods for the prevention and treatment of upper respiratory tract diseases in cats, as well as early differential diagnosis and timely next step treatment in wild protected animals susceptible to infection with the virus. This study further applies the combination of chemical materials and animal disease detection reactions, and also confirms the widespread application value of nanotechnology.

## Data availability statement

The original contributions presented in the study are included in the article/supplementary material, further inquiries can be directed to the corresponding authors.

## Ethics statement

The animal study was approved by this study was approved by the Animal Ethics Committee of Institute of Special Animal and Plant Sciences, Chinese Academy of Agricultural Sciences. The study was conducted in accordance with the local legislation and institutional requirements.

## Author contributions

MY: Data curation, Methodology, Writing – original draft, Writing – review & editing. JS: Software, Writing – review & editing. XZ: Formal analysis, Writing – review & editing. SW: Investigation, Writing – review & editing. CW: Supervision, Writing – review & editing. ZW: Formal analysis, Writing – review & editing. GL: Data curation, Writing – review & editing. LY: Project administration, Writing – review & editing. XS: Data curation, Project administration, Writing – review & editing. YC: Funding acquisition, Project administration, Writing – review & editing. EF: Data curation, Formal Analysis, Writing – review & editing.
